# Addiction and the adrenal cortex

**DOI:** 10.1530/EC-13-0028

**Published:** 2013-07-04

**Authors:** Gavin P Vinson, Caroline H Brennan

**Affiliations:** 1 School of Biological and Chemical SciencesQueen Mary University of LondonLondon, E1 4NSUK

**Keywords:** behaviour, corticosteroids, HPA axis, glucocorticoid

## Abstract

Substantial evidence shows that the hypophyseal–pituitary–adrenal (HPA) axis and corticosteroids are involved in the process of addiction to a variety of agents, and the adrenal cortex has a key role. In general, plasma concentrations of cortisol (or corticosterone in rats or mice) increase on drug withdrawal in a manner that suggests correlation with the behavioural and symptomatic sequelae both in man and in experimental animals. Corticosteroid levels fall back to normal values in resumption of drug intake. The possible interactions between brain corticotrophin releasing hormone (CRH) and proopiomelanocortin (POMC) products and the systemic HPA, and additionally with the local CRH–POMC system in the adrenal gland itself, are complex. Nevertheless, the evidence increasingly suggests that all may be interlinked and that CRH in the brain and brain POMC products interact with the blood-borne HPA directly or indirectly. Corticosteroids themselves are known to affect mood profoundly and may themselves be addictive. Additionally, there is a heightened susceptibility for addicted subjects to relapse in conditions that are associated with change in HPA activity, such as in stress, or at different times of the day. Recent studies give compelling evidence that a significant part of the array of addictive symptoms is directly attributable to the secretory activity of the adrenal cortex and the actions of corticosteroids. Additionally, sex differences in addiction may also be attributable to adrenocortical function: in humans, males may be protected through higher secretion of DHEA (and DHEAS), and in rats, females may be more susceptible because of higher corticosterone secretion.

## Introduction

The purpose of this review is to demonstrate the critical role of the adrenal cortex in addiction and additionally to propose that sex differences in adrenocortical function may contribute to sex differences in addiction. Where it is clear, the sex of experimental animals or of human subjects in the cited studies is stated, although in most cases sex differences were not emphasized.

There is a long history of associating addiction with the adrenal. Indeed, it was well before the adrenocortical hormones were even characterized that morphine toxicity was linked to the adrenal gland. Thus, Lewis (1) and Mackay & Mackay (2) showed that adrenalectomy increased morphine sensitivity in female rats, and chronic treatment with morphine in males or methadone in either sex produces adrenocortical hypertrophy (3, 4). Consequently, there has been interest in the actions of the hormones of the adrenal as possible agents in addiction from the time of their discovery. Treatment with cortisone (the therapeutic corticosteroid of choice at the time) was soon applied in the management of meperidine and morphine withdrawal symptoms in men (5), apparently with beneficial effects, while Lovell associated alcoholism and drug addiction with hypoadrenocorticism (6).

More systematic study then discounted corticosteroids along with other novel ‘cures’ for withdrawal symptoms, and Fraser & Isbell (7) were the first to suggest that in fact withdrawal symptoms (from morphine) in men were associated with eosinopaenia, a measure used at that time to reflect high levels of circulating corticosteroids (8). Eosinophil counts swiftly normalized when morphine was restored. These authors also found that treatment with either cortisone or ACTH shortened the period for development of withdrawal symptoms in men, and therefore, they themselves could be considered a cause (7, 9, 10, 11). Indeed, chronic treatment with corticosteroid can itself lead to later withdrawal symptoms (12).

So there are fundamental questions on the role of corticosteroids in addiction. Is the lower adrenocortical activity in sustained morphine administration, and its elevation when administration ceases, a cause or an effect of addictive responses? Could the drive to addictive drugs actually represent a drive to lower cortisol, with its sequelae? Or is the heightened secretion of corticosteroids in drug withdrawal simply a response to stress? We here argue that the adrenal cortex has a critical role in the acquisition of addiction and also in protection against it.

## The hypophyseal–pituitary–adrenal axis in the brain and addiction

In relation to addiction, far more attention has been paid to hypophyseal–pituitary–adrenal (HPA) components in the brain than to the systemic (i.e. blood-borne) HPA axis. All the components are present in the brain, and, in relation to the hypothesis that the adrenal itself is crucial to addiction, it is important to unravel the relationship between brain and systemic HPA function. This section examines the evidence for brain HPA function in addiction and shows that it is not autonomous, and its function is closely regulated by and linked to the systemic HPA.

### Corticotrophin releasing hormone

Corticotrophin releasing hormone (CRH) is produced in various parts of the brain (13). First, CRH exerts its systemic effects following its release at the median eminence by neuronal tracts that originate in the paraventricular nucleus (PVN) of the hypothalamus. CRH is transported to the corticotrophs of the anterior pituitary via the hypophyseal portal system and then stimulates the secretion of ACTH. ACTH is in turn carried in the general circulation and stimulates the secretion of corticosteroids in the adrenal cortex.

**Figure 1:**
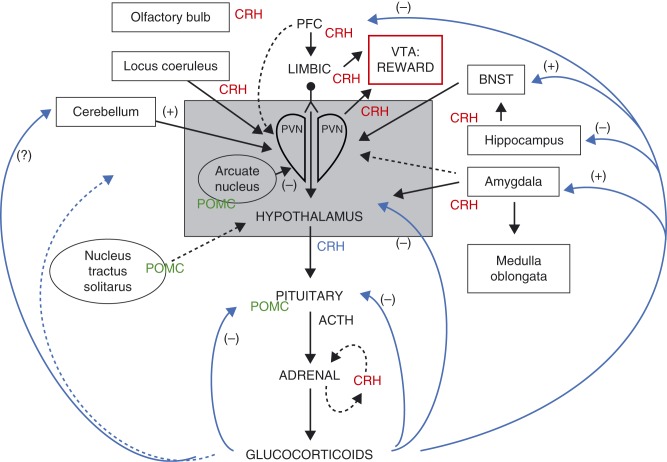
The expanded HPA axis. From (20, 49, 80, 82, 192, 193) and see text. BNST, bed nucleus of stria terminalis; PFC, pre-frontal cortex; PVN, paraventricular nucleus; VTA, ventral tegumental area (associated with reward responses); CRH, corticotrophin releasing hormone; POMC, proopiomelanocortin; +, stimulatory; −, inhibitory. Solid arrows show proven regulation, and dotted arrows show postulated actions. Secreted CRH is indicated in blue lettering, and sites of CRH and POMC signalling are indicated in red and green respectively: here, arrows indicate regulatory pathways that are unquestionably multifactorial but may include actions of CRH and POMC peptides. The inhibitory effect of neural POMC peptides on PVN CRH is particularly interesting, and, by comparison with other systems, might suggest a negative feedback mechanism; however, there is little evidence for reciprocal feedback of CRH on POMC in the brain. Instead, regulation of neural POMC is multifactorial (e.g. (65, 67), and this is primarily linked to its role in energy balance and nutrition, see text. There is, however, much evidence to show the feedback of glucocorticoids on CRH expression in several brain regions. Mostly, this is negative, except in the amygdala, a key region in addiction (19), where it is positive.

In addition, however, CRH, its receptors CRHR1 and CRHR2, and also CRH binding protein (CRH-BP), which modulates CRH actions, are found in other brain locations, where CRH presumably acts primarily as a neurotransmitter. These sites include the cerebrocortex, limbic system, hippocampus, amygdala, locus coeruleus, olfactory bulb and cerebellum (14, 15, 16, 17, 18, 19, 20). While the involvement of such extra hypophyseal CRH with addiction may be independent of the HPA (18, 20), there are certainly pathways through which it contributes to the multifactorial regulation of hypothalamic CRH (Fig. 1).

In the brain, CRH binds to both receptor types, CRHR1 and CRHR2. In addition to CRH itself, both these receptors bind ligands of the urotensin family. The two receptors mediate different responses; CRHR1 agonists produce stress-related responses on which CRHR2 may have less effect, while more potently depressing food intake (21, 22, 23, 24).

There is certainly substantial evidence for the role of CRH in addiction (18, 25), and particularly in reinstatement, but the data are not always consistent. For example, cocaine stimulates the HPA axis through a hypothalamic/CRH-mediated mechanism in male rats (26, 27), and although this is not invariably closely linked to corticosterone (28), both *Crh* mRNA transcription and circulating corticosterone are further increased on cocaine withdrawal (29). In contrast, shock-induced reinstatement of heroin or alcohol seeking clearly depends on CRH, but not on corticosterone, according to some authors (30, 31, 32). Nevertheless, adrenal function is required during cocaine self-administration for subsequent CRH-dependent shock-induced reinstatement to occur (33). The modulator of CRH actions, CRH-BP, is now emerging as an additional factor, although not so widely studied in the addiction field (34, 35). Although both corticosterone and ACTH secretion are increased by acute alcohol exposure, they are inhibited in chronic exposure (36, 37). Neither CRH nor cortisol is implicated in cocaine reinstatement in squirrel monkeys (38).

With specific regard to morphine and the opioids, it is clear that reduced circulating corticosteroid concentrations may be a consequence of opioid inhibition of CRH secretion, acting through μ- and κ-type opioid receptors in the male rat hypothalamus (39, 40, 41). In humans, opioids directly inhibit CRH secretion and the HPA axis, resulting in decreased circulating cortisol. In male rats, the effect is biphasic, with early enhancement of CRH (and the HPA) followed by inhibition after a few days of treatment (41, 42); such responses are affected by stress in male rats (43). Indeed, the evidence suggests that opioidergic mechanisms may at least partially underlie both the behavioural effects of CRH in male rats (44) and also the increase in CRH secretion under conditions of stress. This may not be true in other situations such as the increased HPA activity in adrenalectomized animals (45). This double effect in rats may be because opioids have differential effects on different cell types: they certainly inhibit CRH secretion that is promoted by neurotransmitters (46). The possibly critical involvement of opioids in alcohol addiction in humans (47) has also been shown to be exerted via other than HPA pathways (48).

There are clear differences between the actions of different addictive drugs on *Crh* mRNA transcription in the hypothalamus, and although alcohol acts directly on the PVN, other drugs, including cocaine, nicotine and cannabinoids, activate *Crh* transcription in other brain sites (49). Adrenocortical activity may still be critical, for example in reinstatement of cocaine addiction in male rats (33). Timing of exposure is also significant; early exposure can affect subsequent responses (50), and in male rats, adolescent exposure to alcohol vapour blunts subsequent adult *Crh* transcription response to acute alcohol (51).

The development of specific CRHR1 antagonists has provided more information. CRHR1 blockade inhibits further alcohol drinking in male rats habituated to a high intake (52), and, in conjunction with additional studies using *Crh1* knockout animals, it has been shown that CRHR1 signalling pathways are essential for sensitization to alcohol addiction in male mice (53); a common expression of neuroadaptations induced by repeated exposure to addictive drugs is a persistent sensitized behavioural response to their stimulant properties. These authors also show that acquisition and sensitization are differentially regulated. Acquisition involves the HPA axis and is inhibited by the glucocorticoid blocker mifepristone as well as by CRHR1 blockade, whereas sensitization is unaffected by mifepristone. Pastor *et al*. (53) propose that this suggests a non-hypothalamic CRHR1-linked pathway in sensitization. Different effects were seen in methamphetamine (MA) responses, in which behavioural sensitization measured as increased drug-induced locomotor activity was unaffected in *Crh1* knockouts or by the antagonist CP 154 526 in DBA/2J mice, whereas deletion of *Crh2* attenuated MA-induced behavioural sensitization. Here, an action of endogenous urocortins was suggested, focused in the basolateral and central nuclei of the amygdala (54).

### Proopiomelanocortin

Proopiomelanocortin (POMC) provides, in ACTH and α-melanocyte stimulating hormone (α-MSH), the other components of the HPA axis, and in this context, its primary site of expression and processing is the anterior pituitary and (in rodents) the pars intermedia. POMC is also expressed in brain sites, primarily in projections from the arcuate nucleus of the hypothalamus and from the nucleus tractus solitarius of the brainstem (55, 56, 57). Its primary role in the brain is the generation of α-MSH, which participates in the regulation of food intake and in the production of β-endorphin, pain control. α-MSH acts through two of the melanocortin receptor (MCR) series, MC3R and MC4R, and the latter may also regulate aspects of pain recognition (25, 58).

POMC expression and processing suggests that although ACTH and other POMC products such as β-endorphin can be found in non-hypothalamic regions of the brain or cerebrospinal fluid (59, 60), some may be transported to the brain from the blood (60, 61). From early development, the major adrenocortical-related POMC product in the brain is α-MSH (62), presumably associated with the distribution of the prohormone convertases PC1 and PC2 (63, 64). By far, the major focus of attention in this regard is the role of α-MSH with leptin, ghrelin and agouti protein in the regulation of food intake and energy balance (56, 62, 65, 66, 67, 68).

In addition to its role in energy balance, α-MSH also plays a part in the physiology of addiction, and MC4R, like CRH receptors, respond to morphine (69, 70, 71), and the behavioural effects of morphine or cocaine are modulated by selective MC4R inhibition (72, 73). Additionally, acute alcohol treatment reduced α-MSH expression in hypothalamic and other brain locations in rats, but chronic treatment enhanced it (74).

Of course, POMC processing in relation to addiction cannot be considered purely in terms of its HPA-linked functions. The production of β-endorphin leads inevitably to direct effects on addiction pathways. Its main action is mediated by μ-receptors as are the opiates morphine, heroin and methadone, and in humans, the endogenous opiates are similarly inhibitory on HPA function, although both stimulatory and inhibitory in rats (49, 75).

What has not been clear hitherto is whether the term ‘HPA axis’ can in reality be extended to these components in the brain. In other words, it has been unclear whether, for example, non-hypothalamic CRH provokes synthesis, processing or release of POMC in the brain, but the different locations of the expression of these components may suggest it does not (Fig. 1). Similarly, there has really been no evidence that brain CRH or POMC products have any interaction with the adrenal cortex and the secretion of glucocorticoids, other than via the hypothalamus. On the contrary, it has sometimes been assumed that they do not (e.g. (53)). However, neural glucocorticoid receptor (GR) disruption, including in the PVN, ameliorates the effects of anxiety and also results in heightened HPA activity in male mice (76), consistent with the loss of glucocorticoid inhibition of CRH (20, 77). In contrast, forebrain-specific GR knockout, which does not involve the PVN, increased anxiety behaviour but has the same effect of diminishing glucocorticoid inhibition of CRH in male mice (77). It is clear from this study that the HPA is regulated partly by forebrain GR-mediated inhibition. Accordingly, what needs to be unravelled is the significance of the local brain CRH/POMC components in distinction to that of the systemic HPA, and how independent these systems really are in addiction.

### Interaction between brain CRH and α-MSH

Although the main recognized function of α-MSH in the brain, regulation of food intake and nutrition seems not to be closely related to that of CRH, in fact there is ample evidence of crosstalk between them. Certainly, like the systemic HPA, POMC-processing neurones are activated by stress and play a role in the consequent behavioural response in male rats (78, 79). Furthermore, neuronal POMC-derived peptides regulate hypothalamic CRH and thus ACTH secretion in male and female mice (80). Additionally, α-MSH stimulates *Crh* transcription in the PVN of male rats (81, 82), although, like γ-MSH, it also inhibits interleukin-1β-induced HPA activity, apparently through central MCRs (83). That the circuit connecting brain and systemic HPA is complete is suggested by the finding that glucocorticoids enhance MC4R signalling in a hypothalamic neuronal cell line (84). We can therefore predict the existence of an extended HPA axis in which the same components, CRH, POMC products and corticosteroids as in the classical system, also interact in the brain (Fig. 1) with specific effects on mood and behaviour. The two systems, brain and somatic, interact to the extent that whatever physiological stimuli activate the systemic system, broadly ‘stress’ and the clock, must also have consequences on mood and behaviour.

### Steroids in the brain

The spectrum of structures and functions of neurosteroids is so wide as to form a branch of endocrinology (or at least paracrinology) in its own right. Many are locally synthesized, although usually requiring substrates from non-neural sources. Oestrogens are prominent among these and are produced by aromatase activity in the hippocampus, acting, it is thought, on locally produced C_19_ steroid substrates (85). They have roles in neural plasticity (86) and neuroprotection (85, 87, 88) and regulate the function of other neurally active agents, including neuroprogesterone, which is also synthesized locally (89). There are sex-related differences in the neural responses to oestrogen (90, 91, 92). Oestrogen action in the brain is mediated through classical oestrogen receptors α and β and also through membrane metabotropic glutamate receptors (93, 94). Neuroactive steroids that primarily act through *N*-methyl-d-aspartate or gamma-aminobutyric acid (GABA) receptors include the adrenal androgen DHEA, which as DHEAS conjugate is the most abundant steroid in human plasma (95, 96, 97, 98). DHEA is not secreted by the rat adrenal cortex: its presence and activity in the brain reflect its local synthesis (99). DHEA and pregnenolone, both Δ^5^,3β-hydrosteroids, are also opioid sigma receptor agonists, whereas progesterone, which has the Δ^4^,3-one configuration, is an antagonist (100). Through their sigma-1 agonist actions, pretreatment with DHEA or pregnenolone potentiates cocaine-induced conditioned place preference (CPP) behaviour in mice (100) but attenuates cocaine-seeking behaviour (101). In patients, DHEA and DHEAS are associated with beneficial actions in cocaine withdrawal (102, 103), and the use of DHEA administration to assist opioid withdrawal has been studied, with variable outcomes (104, 105).

Other known neurosteroids include 3α-hydroxy-5α-pregnan-20-one (tertrahydroprogesterone, allopregnanolone, THP) and 3α,21-dihydroxy-5α-pregnan-20-one (tetrahydrodeoxycorticosterone, THDOC), and they are formed in the brain from progesterone and deoxycorticosterone (106, 107). They have anxiolytic, anti-convulsant and sedative activities and are known to be elevated in both plasma and brain in response to ethanol in rats (106, 108). In addition, the HPA axis is under tonic GABA inhibition at the hypothalamic level (75). Importantly, production in the brain of both THP and THDOC depends on precursor steroids of adrenal origin (106).

The corticosteroids themselves have neurological effects, and brain concentrations of corticosterone certainly have relevance to addictive behaviour in male rats (109), and see below. However, the relevance of local brain synthesis of corticosteroids is unclear. Certainly, all the required enzymes of the corticosteroid biosynthetic pathway from cholesterol are present, notably in the hippocampus, together with the StAR protein (110, 111, 112), but their level of production is likely to be low in comparison with concentrations crossing the blood–brain barrier, and they are not thought to be produced in the brain to any great extent (113, 114). Remarkably then, of the known neurosteroids, the corticosteroids may fall into a group of their own being predominantly dependent on an extraneural source: the adrenal cortex.

## The role of the adrenal cortex

### Corticosteroids and mood

Clearly, the role of corticosteroids in addiction cannot be understood without reference to the nature of the psychological and behavioural aspects of the actions of corticosteroids themselves. Almost as the corticosteroids were first characterized, their paradoxical capacity to generate both euphoria and depression in humans has been well known, although poorly understood (115, 116). Changes in mood are a feature of chronic corticosteroid therapy, with mild euphoria in the short term and increases in severity of symptoms associated with depression, or even psychosis in the long-term, and these occur most frequently in women (116, 117, 118, 119, 120), although with large variations in incidence in different studies. Moreover, both cortisol levels and the response to ACTH are higher in depression or depressive episodes (121), and animal experiments show that both of these may be linked to high CRH secretion (29). It has been suggested that corticosteroids may have a role in dopamine-related psychiatric disorders (122), and it has also been speculated that some behavioural features in animals and humans may result from structural or other changes in the brain that corticosteroids may invoke, or at least facilitate (114, 123, 124). Reduction of circulating corticosteroid levels, in combination with other indices, can also be used as a marker for response to anxiolytic therapy (125, 126). It has been postulated that depression in fact reflects GR desensitization, giving rise to impaired glucocorticoid feedback at the hypothalamus, hence increased HPA activity. In this model, one action of antidepressants is thus to resensitize GR transcriptional activity (125), independent of their action on monoamine reuptake, but perhaps involving regulation of steroid elimination from the cell through the multi-drug resistance P-glycoprotein membrane transporter system (127, 128). Together, these studies suggest that corticosteroid-evoked mood changes could be related to behavioural responses to addiction.

### Corticosteroids and addiction

Although the earlier association between the adrenal cortex and addiction is derived largely from circumstantial evidence, there are now data showing a direct causal link. From their experiences with patients receiving chronic steroid treatment, some authors have been willing to label the corticosteroids as drugs of addiction themselves (129, 130, 131, 132, 133, 134), although much of the earlier evidence is based on individual case reports. These findings tend to suggest a close link between corticosteroids and addiction, a concept amply borne out by more recent studies. Alcohol administration induces ACTH secretion and thus adrenocortical stimulation in male rats (106). In habituated men smoking high- but not low-nicotine cigarettes, increased plasma ACTH and cortisol occurs within minutes of smoking (135). Further evidence for the crucial actions of elevated cortisol is given by its association with impaired learning and memory in abstinent cocaine-dependent men and women (136), although higher basal cortisol levels are associated with improved memory performance in healthy controls. These effects on memory apparently reflect the inverted U-shaped cortisol response curve; at low levels, increased cortisol is beneficial to hippocampal cognitive responses, but at higher levels, it is not (137). The degree of stress-induced cortisolaemia and mood negativity is correlated with increased positivity after amphetamine in men and women (138).

Furthermore, much experimental evidence supports the general concept (see Table 1). Male rats too self-administer corticosterone in a manner that suggests some degree of dependence (139, 140). Thus, de Jong *et al*. (141) found that cocaine-induced locomotor sensitization in adrenalectomized male mice was restored by replacement of both adrenaline and corticosterone, and cocaine- or alcohol-induced behaviours in female mice are inhibited in the presence of a GR inhibitor (142). Additionally, if corticosteroid synthesis is blocked, cocaine self-administration also relapses according to some authors (143). Others find the reverse that corticosterone facilitates relapse, although dexamethasone did not, suggesting mineralocorticoid receptor (NR3C2, MR) involvement (144). Such effects, like those of antipsychotic drugs, may be mediated through the mesolimbic dopaminergic system (145, 146). It is striking that dopamine-dependent responses to morphine require glucocorticoid receptors (147).

In experimental animals, the definitive evidence for the pivotal role of the corticosteroids in addiction stems from recent studies in the effects of GR over- and under-expression. Brain-specific GR depletion in mice decreased cocaine self-administration, while corticosterone replacement restored it (148). Specific GR disruption in dopaminoceptive but not dopamine neurones decreased cocaine self-administration (149), whereas GR disruption in either type attenuates cocaine-induced CPP, with no effect on morphine-induced behaviour (150). Morphine-induced CPP depends on hippocampal and nucleus accumbens GR (151). In male mice, overexpression of forebrain GR results in heightened sensitization to cocaine as well as anxiety (152).

There is also evidence of the pivotal role of GR in studies of GR polymorphisms in humans, which have revealed association of particular alleles with the initiation of alcohol abuse in female adolescents (153). These and further experimental data that now link addictive behaviour and symptoms with corticosteroids, particularly in response to cocaine, are summarized in Table 1.

### Sex differences in addiction

The possibility of sex differences in responses to drugs of addiction of brain CRH, POMC, neurosteroids and the HPA axis has not been addressed anywhere in the literature reviewed here. Sometimes, the sex of experimental animals used is not actually given, although this is rare. The impression is that studies are often performed on animals of the same sex – male rats are frequently used – to minimize variance. Yet sex differences in addiction are clear and the extensive evidence has been reviewed in human subjects and in experimental animals. Thus, women are more susceptible to addiction and are at greater risk of relapse than men (154, 155), and female rats are more susceptible than male rats. Substantial evidence links this to gonadal hormones (156).

There is nevertheless good reason to speculate that adrenocortical hormones are involved here as well. Both humans and rats have sex differences in adrenocortical function, and although different in nature, both may contribute to sex differences in addiction.

In humans, differences in circulating cortisol in males and females are marginal at most, though there may be differences in responsiveness to ACTH (96, 157, 158). However, the major product of the gland is in fact DHEA, which is secreted not only as the free steroid, but also, and predominantly, as the sulphate, DHEAS. Plasma concentrations of DHEA and DHEAS in young adult men are about 12 nM and 10 μM respectively, compared with about 8 nM and <7 μM in women, levels decrease with age but the sex differences are maintained (96, 159, 160, 161).

The point is that DHEA has been shown to be protective against drugs of addiction, as previously noted. Evidence from cerebrospinal fluid suggests that adrenal DHEA, and even DHEAS, may reach the brain in significant amounts (162), although how this relates to amounts synthesized within the brain cannot be assessed. Although no sex differences in cerebrospinal fluid were reported, it remains plausible that men receive more DHEA protection to addictive drugs than women (154, 162).

In rats, the situation is different, and there is no significant adrenal secretion of DHEA. However, there is a profound difference in secretion and circulating concentrations of corticosterone (the main glucocorticoid in the rat); adult female adrenals are nearly twice the size of males; and output of corticosterone is proportionately greater (163, 164, 165, 166). Although as noted earlier, DHEA is synthesized in the rat brain, there is no sex difference, and brain concentrations are similar in males and females (167). Accordingly, in the rat, it is plausible that heightened sensitivity to addictive drugs in females is associated with the higher circulating levels of corticosterone.

### The adrenal, addiction and the clock

If it is the adrenal gland itself that is critical for HPA-modulated addictive processes, then other factors that are instrumental in generating adrenocortical responses may be expected to interact with addiction. Of the physiological stimuli that stimulate the adrenal cortex, stress is the most prominent and relevant. However, an equally potent regulator of the adrenal cortex is the clock.

That stress, however defined, facilitates addiction in both patients and animal models is well understood (168, 169, 170, 171, 172). It is deeply interesting to note that clock time too has its effect on addictive craving and behaviours, although this literature generally has little reference to the HPA, but has been focused on the pineal and melatonin in the brain of male mice (173), or, primarily, on clock genes. Periodicity in PER1 and cocaine sensitivity are associated in male rats and mice of various strains (174), drug reinstatement can be suppressed by photoperiod in male rats (175), and clock gene variants are associated with cocaine sensitization in *Drosophila* (176) as with addiction in mice (sex not given) (177) and in humans, according to some authors (178, 179, 180, 181) but not all (182). In men, alcohol consumption over a 26-hour period affected neither melatonin nor the cortisol secretory diurnal variation (183, 184).

### Autonomy of the adrenal

One feature of adrenocortical function that is hardly considered, in relation to addiction or anything else, is that mechanisms exist whereby the secretion of glucocorticoid appears to be regulated in part by local stimuli. CRH is notable among these. The relationship between the functions of hypothalamic CRH and CRH formed locally in the adrenal is currently obscure. That the adrenal gland of various species may secrete CRH from the medulla in response to splanchnic nerve stimulation has been shown, as has the direct stimulatory effect of CRH on corticosteroid secretion (185, 186, 187, 188). How does adrenal CRH vary with addiction? This is a topic for the future.

## Conclusion

There is a clear pattern in the relationship of HPA activation to the development of addictive behaviours in response to quite different drugs. What is it they all have in common? Is there a unifying pathway that in so many cases leads to what may sometimes appear to be an addiction to the adrenal cortex and the secretion of glucocorticoids?

One point is becoming clear: CRH and POMC at different brain sites have clear functional links with the classical HPA (Fig. 1), and together, they may play similar roles in the adaptation that underlies addictive behaviour. They may be considered in the context of addiction as an expanded HPA, of which the terminal, and crucial, component is the adrenal cortex itself.

The evidence for the key importance of the adrenal cortex and glucocorticoids in behaviour and symptoms in drug withdrawal and reinstatement seems conclusive. Therapeutic control of glucocorticoid secretion or inhibition of glucocorticoid action at its receptor may be important future developments (148, 189) in what otherwise is a bleak therapeutic landscape (48, 189, 190, 191).

## Figures and Tables

**Table 1:** Glucocorticoids and addiction. All the direct experimental evidence for the essential role of glucocorticoids has been obtained in experimental animals, as illustrated here. Evidence from the human species is indirect and circumstantial but appears to support the general conclusion that glucocorticoids, regulated by an expanded HPA axis, underlie the important features of addiction.

**Species**				**Effects**	**Reference**
Rats^a^	Corticosterone	Administration	Up to 100 μg/ml in drinking water	Induced corticosterone self administration	139
			Up to 50 μg/animal per day	Induced corticosterone self administration	140
			Up to 0.8 mg/kg implant	Induced corticosterone self administration	194
		Stress induced	Novel environment	Induced amphetamine self administration	195
			Immobilisation	Impaired HPA feedback in cocaine habituated animals	28, 29
		Synthesis blocked	By metyrapone 50 mg/kg	Reduced psychomotor effects of cocaine, and reduced reinstatement	196
			By metyrapone 100 mg/kg	Reduced psychomotor effects of cocaine, and reduced reinstatement	143
			By metyrapone synergistic with benzodiazepine agonist oxazepam; up to 45 mg/kg: 20 mg/kg i.p.^c^	Reduced psychomotor effects of cocaine, and reduced reinstatement	197
			By adrenalectomy^d^	Cocaine reinstatement reduced, restored by corticosterone replacement	198
				Reduced Fos response to dopamine agonist, enhanced dopamine response to cocaine	199
				Decreased cocaine-induced locomotor sensitisation	200
			With corticosterone hemi-succinate replacement; up to 3 mg/kg implant	Restoration of cocaine-induced sensitisation	200
		Levels in blood		Unrelated to high or low responder to cocaine classification	201
		Levels in brain		Related to high or low responder to cocaine classification	109, 202
Mice^b^	GR		Antagonist, mifepristone 30 mg/kg i.p. (or, less effective, MR antagonist spironolacetone 20 mg/kg i.p.)	Reduced cocaine induced reinforcement	203
		Selective GR depletion	In brain	Decreased sensitisation to cocaine self administration	148
			In brain	Selective reduced glutamate receptor subunit, and enkephalin response to cocaine, no effect on neuropeptide or dopamine receptor response	204
			In dopaminoceptive neurones	Decreased cocaine self administration	149
			In dopaminoceptive or dopamine neurones	Decreased cocaine induced CPP	150
		Selective GR overexpression	In forebrain	Increased cocaine sensitisation	152
	Adrenalectomy		With corticosterone (20 mg in pellets) and adrenaline (5 μg/kg s.c.) replacement^e^	Synergistic actions on restoration of cocaine induced locomotor sensitisation	141

a Sprague Dawley strain except where stated

b Original strain usually C57B/6

c Wistar rats

d Long Evans rats

e DBA/2 Rj strain
